# Oropharyngeal Shedding of Gammaherpesvirus DNA by Cats, and Natural Infection of Salivary Epithelium

**DOI:** 10.3390/v14030566

**Published:** 2022-03-09

**Authors:** Elizabeth C. Rose, Tiffany Y. Tse, Andrew W. Oates, Ken Jackson, Susanne Pfeiffer, Shannon L. Donahoe, Laura Setyo, Vanessa R. Barrs, Julia A. Beatty, Patricia A. Pesavento

**Affiliations:** 1 Department of Pathology, Microbiology, and Immunology, School of Veterinary Medicine, UC Davis, Davis, CA 95616, USA; ecrose2@ncsu.edu (E.C.R.); tytse@ucdavis.edu (T.Y.T.); andrew.oates@colostate.edu (A.W.O.); kajackson@ucdavis.edu (K.J.); papesavento@ucdavis.edu (P.A.P.); 2 Department of Clinical Sciences, College of Veterinary Medicine, North Carolina State University, Raleigh, NC 27606, USA; 3 Department of Microbiology, Immunology and Pathology, College of Veterinary Medicine and Biomedical Sciences, Colorado State University, Fort Collins, CO 80523, USA; 4 Jockey Club College of Veterinary Medicine and Life Sciences, City University of Hong Kong, Hong Kong, China; susanne.pfeiffer@cityu.edu.hk; 5 Sydney School of Veterinary Science, Faculty of Science, University of Sydney, Sydney, NSW 2006, Australia; shannon.donahoe@sydney.edu.au (S.L.D.); laura.setyo@uqconnect.edu.au (L.S.); vanessa.barrs@cityu.edu.hk (V.R.B.); 6 Department of Pathology and Infectious Diseases, School of Veterinary Medicine, Faculty of Health and Medical Sciences, University of Surrey, Guildford GU2 7AL, UK; 7 Department of Veterinary Clinical Sciences, Jockey Club College of Veterinary Medicine and Life Sciences, City University of Hong Kong, Hong Kong, China; 8 Centre for Animal Health and Welfare, City University of Hong Kong, Hong Kong, China

**Keywords:** feline, gammaherpesvirus, oncogenesis, shedding, transmission, carcinoma

## Abstract

*Felis catus* gammaherpesvirus-1 (FcaGHV1), a novel candidate oncogenic virus, infects cats worldwide. Whether the oropharynx is a site of virus shedding and persistence, and whether oronasal carcinomas harbor FcaGHV1 nucleic acid were investigated. In a prospective molecular epidemiological study, FcaGHV1 DNA was detected by cPCR in oropharyngeal swabs from 26/155 (16.8%) of cats. Oropharyngeal shedding was less frequently detected in kittens ≤3 months of age (5/94, 5.3%) than in older animals; >3 months to ≤1 year: 8/26, 30.8%, (*p* = 0.001, OR 7.91, 95% CI (2.320, 26.979)); >1 year to ≤6 years: 10/20, 50%, (*p* < 0.001, OR 17.8 95% CI (5.065, 62.557)); >6 years: 3/15, 33% (*p* = 0.078). Provenance (shelter-owned/privately owned) was not associated with shedding. In situ hybridization (ISH) identified FcaGHV1-infected cells in salivary glandular epithelium but not in other oronasal tissues from two of three cats shedding viral DNA in the oropharynx. In a retrospective dataset of 11 oronasopharyngeal carcinomas, a single tumor tested positive for FcaGHV1 DNA by ISH, a papillary carcinoma, where scattered neoplastic cells showed discrete nuclear hybridization. These data support the oronasopharynx as a site of FcaGHV1 shedding, particularly after maternal antibodies are expected to decline. The salivary epithelium is identified as a potential site of FcaGHV1 persistence. No evidence supporting a role for FcaGHV1 in feline oronasal carcinomas was found in the examined tumours.

## 1. Introduction

Gammaherpesviruses are a subfamily of Herpesviridae that have co-evolved over millennia with a diverse range of mammals. The most extensively studied gammaherpesvirus, Epstein–Barr virus (EBV), is a very common, innocuous part of the human virome in all but a minority of people where EBV infection causes infectious mononucleosis, nasopharyngeal carcinoma, B-cell lymphoma and other malignancies [1]. Factors that precipitate gammaherpesvirus-associated diseases are incompletely understood, but include compromised host immunity in a natural host or “jumping” of a virus from a well-adapted host to a closely related but non-adapted host. Examples of the former include some human AIDS-associated cancers [2], and, of the latter, malignant catarrhal fever (MCF) caused by a cluster of host-adapted MCF viruses that infect non-adapted but related species [3].

*Felis catus* gammaherpesvirus 1 (FcaGHV1) is one of several novel gammaherpesviruses recently identified in animals [4,5,6,7]. Understanding whether or when such viruses are pathogenic is complicated by the likely sporadic and diverse nature of any associated disease, coupled with high rates of infection among clinically normal animals. Epidemiologic studies of FcaGHV1 report that <6% to 23.6% of domestic cats worldwide test positive for circulating FcaGHV1 DNA (“DNAemia”) using virus-specific qPCR [8,9,10,11,12,13]. Serologic data suggest higher rates of exposure than indicated by molecular virus detection [14], consistent with the natural history of gammaherpesviruses where strategies of viral persistence, such as low-copy epithelial replication and an armament of immune evasion strategies, uncouple viremia and antibody response. Given the frequent detection of FcaGHV1 in healthy cats, it is reasonable to assume that asymptomatic infection is common, with host immunity expected to eliminate the virus from all but a small reservoir of persistently infected cells.

On the basis of genomic similarities to human, mouse, and primate gammaherpesviruses, we hypothesized that oropharyngeal tissues are a site of FcaGHV1 persistence and shedding. EBV, for example, is capable of infecting a variety of cell types, but epithelial cells are a major cellular target, and virus shed in the saliva of healthy carriers supports a role for epithelial cells in the amplification of virus during persistent infection [15]. Epithelial cells are also implicated as reservoirs of viral persistence in murine gammaherpesvirus, MHV-68, where a tropism for salivary gland tissue and shedding of virus into saliva facilitates transmission [16,17]. We have investigated whether FcaGHV1 DNA can be detected in secretions and cells shed in the oropharyngeal cavity. In cats where the virus was detected by PCR, we used in situ hybridization (ISH) to investigate the cellular target(s) of viral infection/persistence.

If the oronasal cavity is a site of persistence, oncogenesis is a potential outcome. Long-term exposure of the oronasal mucosa to EBV is proposed as a risk factor for EBV-associated nasopharyngeal carcinoma (NPC) [16] and lymphoepithelioma-like carcinoma (LELC), a poorly differentiated carcinoma arising in several locations including the pharynx and salivary glands [18]. The consistent presence of EBV within transformed cells is a hallmark of these cancers [19]. We used a combination of PCR and ISH to determine whether FcaGHV1 DNA could be detected in neoplastic cells from oronasal epithelial cancers arising in cats.

## 2. Materials and Methods

### 2.1. Ethics Statement

Samples were obtained at routine unrestricted necropsy with written owner consent or were residual diagnostic samples used with consent from the shelter.

### 2.2. Samples

Oropharyngeal (OP) samples were obtained from shelter-housed cats using sterile swabs during a routine examination at shelters in California. Swab tips were placed in 100 µL of Zymo DNA/RNA Shield (Zymo Research, Irvine, CA, USA) and stored at −80 °C. OP swab samples were similarly obtained from privately-owned cats submitted for routine necropsy at UC Davis School of Veterinary Medicine. In addition, for each necropsy case, the following tissues were formalin-fixed and paraffin-embedded (FFPE): tongue, tonsil, labial oral mucosal, salivary gland, pharyngeal mucosa, nasal epithelium/conchae. Age, either documented or estimated, was recorded for each cat. Historic FFPE biopsy material from carcinomas of the feline oronasal cavity was identified and retrieved from the histopathology database at UC Davis.

### 2.3. DNA Extraction and PCR Testing

DNA was extracted from swabs immediately after thawing using the QiaAMP DNA Micro Kit (Qiagen, Germantown, MD, USA). DNA was extracted from frozen tissues and from 25 µm scrolls of FFPE tissue after being deparaffinized in xylene and rehydrated with 100%, 90% and 70% ethanol, using a commercial kit (DNeasy^®^ Blood & Tissue Kit, QIAGEN, Hilden, Germany). DNA samples testing negative for feline glyceraldehyde-3-phosphate dehydrogenase (GAPDH) using conventional PCR (cPCR) were excluded [20]. A cPCR amplifying a 164 bp fragment of the FcaGHV1 glycoprotein B (gB) gene was performed as described previously [21].

The 25-µL reactions contained HotStarTaq Plus Master Mix (Qiagen), 2.5 µL of extracted DNA, 0.2 µM concentrations for each primer GH-3F 5′-TGACATGTAACGCAGTCTATG-3′ and GH-3R 5′-TCTGTGCATGATTCGTTCCAT-3′. The mixtures were amplified with an initial denaturation at 95 °C for 5 min followed by 40 cycles at 94 °C for 30 s, 50 °C for 30 s, and 72 °C for 30 s. There was a final extension at 72 °C for 10 min.

### 2.4. In Situ Hybridization

FFPE OP tissues from three cases testing positive for FcaGHV1 DNA on OP swabs, and three testing negative, were processed for ISH, as described previously (Aghazadeh et al. 2018) [21]. Briefly, colorimetric ISH was performed manually on 5 µm sections of FFPE tissue on Superfrost Plus slides (Fisher Scientific, Pittsburgh, PA, USA) using the RNAscope 2.5 Red assay kit (322360, Advanced Cell Diagnostics, Inc., Hayward, CA, USA) and the ISH probe V-FcaGHV1-ORF50 (510481, ACD). Each section was pretreated with heat and protease prior to probe hybridization for 2 h at 40 °C. Negative controls used for validation of signal included a bacterial gene DapB probe and an uninfected animal. Slides were counterstained with hematoxylin and mounted with EcoMount (Biocare Medical, Concord, CA, USA). Slides were digitized using an Olympus VS120 scanner and a 40× objective with bright field illumination.

### 2.5. Statistical Analysis

Statistical analysis was performed using IBM SPSS Statistics for Windows, Version 26.0 (IBM Corp, Armonk, NY, USA). Age was categorized as ≤3 months (Age Group 1), >3 months and ≤1 year (Age Group 2), >1 year and ≤6 years (Age Group 3), >6 years (Age Group 4). Distributions were tested, and descriptive statistics were derived for the continuous variable Age, and the categorical variables Age Group, Provenance (shelter-owned or privately-owned) and OP Swab PCR Result (positive or negative). Frequencies of OP Swab PCR Results were calculated for all Age Groups. Univariable associations between the categorical variables Age Group and Provenance with OP swab PCR Result were analysed using Fisher’s Exact Test with post-hoc pairwise Fisher’s Exact Tests with Bonferroni correction. Odds ratios were calculated to measure the associations between categorical variables. The effect of Age and Provenance combined as risk factors for a positive OP Swab Result was investigated using a multivariate model. Statistical significance was set at *p* ≤ 0.05.

## 3. Results

### 3.1. Oropharyngeal Shedding of FcaGHV-1

OP swabs were available from 155 cats with a median age of 3 months (range 1 to 240 months, interquartile range [IQR] 11 months). Of these, 137 were shelter-owned (median age 2 months, range 1 to 168 months, IQR 4 months), and 18 were privately-owned (median age 84 months, range 12 to 240 months, IQR 69 months).

Overall, OP shedding was detected in 26/155 cats (16.8%). There was no difference in the frequency of OP shedding between shelter-owned cats (16.8%) and privately-owned cats (16.7%; Fisher’s Exact Test, *p* = 1.00). The frequency of OP shedding stratified by Age Group is presented in Table 1. The frequency of OP shedding was much lower in cats up to the age of 3 months than in older cats (Table 1). In cats over 3 months, OP shedding was detected in 34.4% (21/61). The analysis of Age and Provenance together as risk factors for OP shedding was not performed as the assumptions for a multivariate model were not met.

### 3.2. Persistence of FcaGHV-1 in Salivary Gland Epithelium

**Figure 1:**
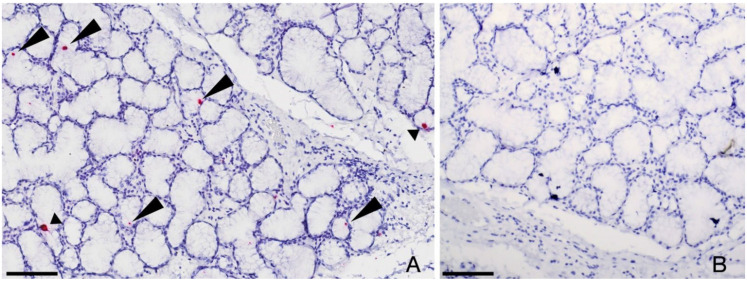
In situ hybridization to detect FcaGHV1 DNA in feline salivary gland. (**A**) FcaGHV-1 probe hybridization is detected within the nuclei of scattered glandular epithelial cells of the salivary gland (arrowheads). (**B**) Using an unrelated probe (DapB), no hybridization is detectable in a duplicate slide (cut in series). Bars 100µm

Two of three cases testing positive for FcaGHV1 DNA on PCR of OP swabs also tested positive on ISH, with a clear, repeatable nuclear signal detected in scattered salivary epithelial cells (Figure 1). This same result was obtained after repeating the ISH procedure on two additional sections from each case. No other sections of oropharyngeal tissues on the same, or other slides (tongue, tonsil, labial oral mucosal, pharyngeal mucosa, nasal epithelium/conchae tissues) from these cases tested positive by ISH. All three cases testing negative for FcaGHV1 DNA on PCR of OP swabs and the remaining PCR positive case, tested negative on ISH on all sections examined.

### 3.3. FcaGHV1 Sequence Not Detected in Neoplastic Cells from Oronasal Carcinomas

**Figure 2:**
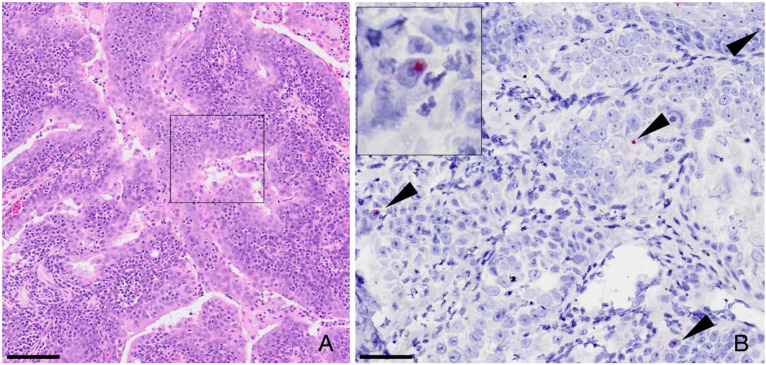
In situ hybridization, papillary carcinoma in a domestic cat. (**A**) Fronds of neoplastic epithelium characteristic of a papillary carcinoma, hematoxylin and eosin. Bar 100µm (**B**) Among eleven tumors tested in this single case, there was rare probe hybridization in scattered nuclei of neoplastic cells (arrowheads and inset). Bar 50µm

Of 11 FFPE oronasopharyngeal carcinoma biopsies (two nasal adenocarcinomas, two papillary carcinomas, four salivary adenocarcinomas, three squamous cell carcinomas), a single sample, a salivary adenocarcinoma, tested positive for FcaGHV1 DNA by PCR. Virus-positive cells were not identified by ISH in sections from the PCR positive case. All 11 tumours were investigated using ISH, regardless of their PCR status. Hybridization was detected in rare, scattered nuclei of individual tumor cells in a single PCR-negative case of nasopharyngeal papillary carcinoma (Figure 2). In addition, ISH-positive cells were also detected in remnant, normal salivary glandular epithelium from the same case that had been processed, incidentally, on the same slide. The pattern of staining in the salivary gland was the same as that described in 3.2 above. The other nine tumours were negative for FcaGHV1 by both PCR and ISH.

## 4. Discussion

This study demonstrates for the first time that FcaGHV1 DNA is shed in the oropharynx of naturally infected cats. Viral shedding in oronasal and other body fluids is reported for other gammaherpesviruses including EBV, Kaposi’s sarcoma herpesvirus, MHV-68, EHV-2 [22] and MCF viruses ovine herpesvirus-2 and alcelaphine herpesvirus-1 (AlHV-1) [3,23,24]. Oropharyngeal shedding of FcaGHV1 implies that FcaGHV1 can be transmitted in saliva and, considering the natural behaviour of cats, that mutual grooming or, as mooted previously, biting are plausible routes of virus transmission [11].

We have shown that salivary epithelium is a target of FcaGHV1 infection using ISH on serial sections, whereas no other infected cell types were identified despite sampling of many oropharyngeal sites. Hence a role for salivary epithelium as a source of virus shed into saliva is possible. Given the small number of infected cells identified, we cannot rule out the presence of virus-infected cells in the other sites sampled. FcaGHV1-infected circulating B and T lymphocytes have previously been described in natural FcaGHV1 infection [25], but much remains to be learned about the cellular targets and reservoirs of this and many other gammherpesviruses. Consensus is lacking even for EBV shedding, although a model where small clusters of tonsillar epithelial cells intermittently amplify virus shed from plasma cells migrating through Waldeyer’s ring is well-supported [15].

FcaGHV1 DNA shedding was commonly detected in our study, with one in three cats over 3 months of age testing positive for virus DNA on OP swabs. Although not directly comparable, a previous study of cats from the same region reported 14/50 (28%) tested positive for FcaGHV1 DNA in blood using virus-specific qPCR [4]. Our data provide a minimum estimate of the frequency of OP shedding of FcaGHV1, since virus could be shed intermittently and cPCR lacks sensitivity compared with FcaGHV1-specific qPCR, which is capable of detecting single-digit copy numbers [4].

An age-related pattern of OP shedding was observed, where the odds of virus detection were around 8 and 18 times higher in cats up to one year-, and up to 6 years- of age respectively, than in kittens up to 3 months of age. The small sample of cats over 6 years old likely precluded the demonstration of a significantly higher shedding frequency in that age group compared to kittens. The observed pattern of OP shedding is consistent with perinatal primary infection by FcaGHV1 being a common infection route, and an onset of virus shedding around the time that maternal antibodies are expected to wane from 6 to 16 weeks [26]. For EBV, age at primary infection varies widely. In regions where early childhood infection is common, infants become infected from 8 months of age, coinciding with maternal antibody decline [27]. In high-income countries, primary infection is increasingly delayed until adolescence based on seroprevalence data and an increasing frequency of infectious mononucleosis caused by primary EBV infection [28,29,30]. Much less is known about the age of natural infection with animal gammaherpesviruses. In wildebeest, the natural host for AlHV-1, primary infection is established in utero or the immediate postpartum period [31].

No evidence to support an association between FcaGHV1 and oronasal carcinomas was found. In EBV associated nasopharyngeal carcinoma, there is widespread hybridization to the EBV genome in the nuclei of neoplastic epithelial cells [16]. The consistent presence of viral DNA in a tumour implies that that the virus is playing a role in the persistence of the tumour cells [32]. In contrast, FcaGHV1 DNA was only detected in a few, scattered neoplastic cells in a single tumour. The negative PCR result from the same tumour can be explained by the capacity of ISH to detect individual cells positive for viral DNA, whereas such cells may not be present in the tumour DNA undergoing PCR, or the virus load in tumour DNA may be below the limit of PCR detection. Bidirectional discordancy was noted with one salivary adenocarcinoma testing virus positive by PCR but not by ISH. This suggests that although FcaGHV1 DNA was detected in the tumour sample by PCR, virus-containing cells were not sampled for ISH, which is conceivable given that far fewer cells are sampled for ISH than for PCR. Hence the cellular location of virus DNA in the PCR-positive salivary adenocarcinoma is not known, and it may even have been detected from blood circulating through the tissue. We recognize that only a limited number of tumours were tested here, only two of which were homologs of the human papillary-type nasopharyngeal carcinoma that is caused by EBV [33]. Hence, we cannot exclude a role for FcaGHV1 in specific subsets of oronasal carcinomas in cats.

## Figures and Tables

**Table 1:** Oropharyngeal shedding of FcaGHV1 DNA by cats in different age groups.

Age Group	Oropharyngeal Swab PCR Result		Comparison of Shedding between Age Groups *p*-Value, Odds Ratio (OR), 95% Confidence Interval (CI)		
	PCR Positive/Number Tested	Frequency of Shedding (%)	>3 Months and ≤1 Year	>1 Year and ≤6 Years	>6 Years
≤3 months	5/94	5.3	*p* = 0.001 * OR 7.91 95% CI (2.320, 26.979)	*p* < 0.001 * OR 17.8 95% CI (5.065, 62.557)	*p* = 0.078
>3 months and ≤1 year	8/26	30.8	-	*p* = 0.231	*p* = 0.716
>1 year and ≤6 years	10/20	50	*p* = 0.231	-	*p* = 0.089
>6 years	3/15	33.3	*p* = 0.716	*p* = 0.089	-

* Denotes significance; *p* ≤ 0.05.
